# A Silent Exonic Mutation in a Rice Integrin-α FG-GAP Repeat-Containing Gene Causes Male-Sterility by Affecting mRNA Splicing

**DOI:** 10.3390/ijms21062018

**Published:** 2020-03-16

**Authors:** Ting Zou, Dan Zhou, Wenjie Li, Guoqiang Yuan, Yang Tao, Zhiyuan He, Xu Zhang, Qiming Deng, Shiquan Wang, Aiping Zheng, Jun Zhu, Yueyang Liang, Huainian Liu, Aijun Wang, Lingxia Wang, Ping Li, Shuangcheng Li

**Affiliations:** 1 State Key Laboratory of Crop Gene Exploration and Utilization in Southwest China, Sichuan Agricultural University, Chengdu 611130, China; zouting@sicau.edu.cn (T.Z.);; 2 State Key Laboratory of Hybrid Rice, Rice Research Institute, Sichuan Agricultural University, Chengdu 611130, China

**Keywords:** RNA splicing, PL1, rice (*Oryza sativa* L.), anther, pollen, male sterility, integrin-α FG-GAP repeat-containing protein

## Abstract

Pollen development plays crucial roles in the life cycle of higher plants. Here we characterized a rice mutant with complete male-sterile phenotype, *pollen-less 1* (*pl1*). *pl1* exhibited smaller anthers with arrested pollen development, absent Ubisch bodies, necrosis-like tapetal hypertrophy, and smooth anther cuticular surface. Molecular mapping revealed a synonymous mutation in the fourth exon of *PL1* co-segregated with the mutant phenotype. This mutation disrupts the exon-intron splice junction in *PL1*, generating aberrant mRNA species and truncated proteins. *PL1* is highly expressed in the tapetal cells of developing anther, and its protein is co-localized with plasma membrane (PM) and endoplasmic reticulum (ER) signal. *PL1* encodes an integrin-α FG-GAP repeat-containing protein, which has seven β-sheets and putative Ca^2+^-binding motifs and is broadly conserved in terrestrial plants. Our findings therefore provide insights into both the role of integrin-α FG-GAP repeat-containing protein in rice male fertility and the influence of exonic mutation on intronic splice donor site selection.

## 1. Introduction

Pollen, as the male gametophyte, plays key roles in flowering plant fertilization for giving rise to fruits and seeds [1]. Pollen development begins with pollen mother cells (PMCs) that differentiated from sporogenous cells in developing anthers [2]. After two steps of meiosis, PMCs develop into microspores [3]. At this stage, microspores are surrounded with a thin primexine, and the depositions of sporopollenin precursors on undulated PM for exine formation initiate thereafter [4,5]. As the microspores go through vacuolation and two rounds of mitosis afterwards, the formation of mature pollen grains, which are coated by two-layered exine and fulfilled of inclusions, is completed [6,7,8].

The developmental processes of pollen occur inside of the anther, which consists of four somatic cell layers, namely, the tapetum, middle layer, endothecium and outer epidermis [6,9,10]. The tapetum is the innermost cell layer of the anther wall and directly contacts with the developing microspores and secrets enzymes, nutrition, sporopollenin precursors and developmental signals [11,12]. During the late pollen development, tapetal cells trigger an apoptosis-like programmed cell death (PCD), which is pivotal for viable pollen formation [13,14]. In addition, as a skin of anther, cuticle locates on the surface of anther epidermis and acts as a barrier to protect pollen development from external biotic or abiotic stress [15,16].

Several genes involved in these processes have been identified. For example, during meiosis, the tapetal cells are highly vacuolated in *undeveloped tapetum 1* (*udt1*) mutant [17]. Mutant of *Tapetum Degeneration Retardation* (*TDR*) had incorrect degeneration of tapetum and middle layer, and its microspores rapidly degraded after being released from the tetrads, resulting in complete male-sterility [18]. Loss-of-function of *Eternal Tapetum 1* (*EAT1*) causes delayed PCD of tapetal cells and aborted pollen development [19]. These three genes encode basic helix–loop–helix (bHLH) transcription factors. TDR and EAT1 directly regulate expression of cysteine protease and aspartic protease encoding genes, respectively, to control the timing of tapetal PCD [18,19]. Besides, another bHLH-like transcription factor, TDR Interacting Protein 2 (TIP2), is also involved in developmental regulation of tapetum [20]. Both TIP2 and TDR are located upstream of EAT1, and they are able to regulate *EAT1* transcription by forming a heterodimer that binds to the promoter of *EAT1* [20,21]. *Persistent Tapetal Cell 1* (*PTC1*) encodes a putative PHD-finger transcription factor that also interacts with TIP2 [22]. Mutation of *PTC1* leads to abnormal tapetal proliferation and defective formation of Ubisch bodies and pollen wall [23]. 

In addition to the role of tapetum PCD controlling, the tapetal development related transcription factors also regulate the expression of key genes in secondary metabolism and transport of materials to affect the formation of pollen and pollen wall. The outer pollen wall, exine, is mainly composed of sporopollenin [5]. In rice, several genes involved in sporopollenin precursors synthesis and shipping have been found, such as *CYP703A3* [24], *CYP704B2* [25], *Acyl-CoA Synthetase 12* (*OsACOS12*) [26,27], *Polyketide synthase 1/2* (*OsPKS1/2*) [28,29,30,31], *Tetraketide α-pyrone reductase 1* (*OsTKPR1*) [31,32], *ATP Binding Cassette G 15* (*OsABCG15*) [16,33,34], and *OsC4/6* [35]. Among them, CYP703A3, CYP704B2, OsACOS12, OsPKS1/2 and OsTKPR1 may form a sporopollenin metabolon similar to their orthologues in *Arabidopsis* [5,8,15,31,32,36]. OsABCG15 and OsC6 function in transporting of sporopollenin precursors from tapetum to pollen surface for exine formation [16,34,35]. The expressions of *CYP703A3* and *OsC6* are directly regulated by TDR [24,35]. The transcript levels of *CYP704B2*, *OsC4* and *OsC6* are reduced in *ptc1* mutant [22,23]. 

Normal pollen development requires correct gene expression. In eukaryotes, pre-mRNA splicing, including the removal of introns from nascent pre-mRNA transcripts and the ligation of exons, is fundamental for gene expression [37,38]. The specific nucleotide sequences within the borders between introns and exons of pre-mRNA determine where the splicing occurs [39]. The accuracy of splice sites recognition is precisely controlled [40]. It is therefore possible that any variation in the nucleotide sequences of the splice site causes erroneous gene transcription and translation, which in turn affects protein function and even abnormal development of the organism [41,42,43]. In many cases, mutations in the intronic splice sites result in aberrant mRNA splicing. For example, in rice, the *Waxy* (*Wx*) gene encodes a granule-bound starch synthase, which regulates the amylose content and determines the eating and cooking quality [44]. In *indica* rice that contains high endosperm amylose level, *Wx* with a normal sequence has correct mRNA transcripts, whereas the *Wx* in *japonica* rice shows a single base change at the 5′ splice site (splice donor site) of the first intron, representing a larger mRNA with retention of the first intron, which reduces the levels of Wx protein and endosperm amylose [44,45,46]. A mutation at the 3′ splice site (splice acceptor site) of a intron in *MUTANT snc1-ENHANCING* (*MUSE*), an *Arabidopsis* RNA polymerase III subunit, gives rise to intronic retention and partial loss-of-function of MUSE, leading to pleiotropic defects in vegetative development [47]. The *Arabidopsis pasticcino 2-1* (*pas2-1*) mutant harbors a point mutation in the splice donor site of the eighth intron of *PAS2*, which causes a longer mature mRNA with an insertion of an intron and exhibits defective organogenesis with fused-organs [48,49]. A combined missplicing and missense point mutation at 3′ terminal of exon 8 in *Arabidopsis* 2-phosphoglycolate phosphatase 1 gene (*AtPGLP1*) resulted in aberrant splicing of this gene and the consequent conditional lethal phenotype [50]. However, the events of exonic mutations affecting mRNA splicing are rarely reported, especially in the process of plant male gametophyte development. 

In this study, we described the identification of a novel exonic mutation that induced suppressed selection of normal splice donor site and splicing aberrations and is responsible for the abnormal tapetum and pollen development and defective anther cuticular formation in rice *pl1* mutant.

## 2. Results

### 2.1. Isolation of the pl1 Mutant

**Figure 1:**
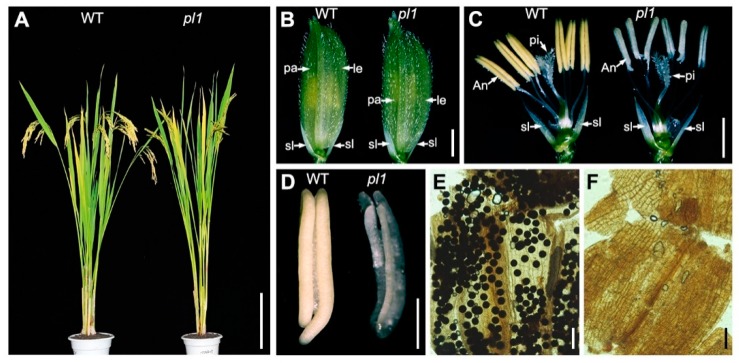
Phenotypic comparison between the WT and *pl1* mutant. (**A**) Plant phenotype of the WT and *pl1* mutant at grain-filling stage. (**B**) Spikelet of the WT and *pl1* mutant at heading stage. le, lemma; pa, palea. (**C**) Spikelet of the WT and pl1 mutant after removing of the lemma and palea. An, anther; pi, pistil; sl, sterile lemma. (**D**) Anther of the WT and *pl1* mutant. (**E**) I2 -KI staining of WT pollen grains at maturing stage. (**F**) I2 -KI staining of *pl1* pollen grains at maturing stage. Bars = 20 cm in (**A**), 2 mm in (**B**) and (**C**), 1 mm in (**D**), and 100 µm in (**E**) and (**F**).

To identify new genes that affect microsporogenesis in rice, we screened an ethyl methane sulfonate (EMS)-induced mutant library in the background of *indica* rice cultivar 9311 and obtained the *pl1* mutant. No obvious morphological differences were found between the wild-type 9311 (WT) and *pl1* plants during the vegetative growth stage (Figure 1A). At the heading stage, though *pl1* plants had normal inflorescence morphology (Figure 1B), their anthers were white and thinner compared with those of the WT (Figure 1C,D). A closer examination through I_2_-KI staining revealed that pl1 anthers were devoid of pollen grains (Figure 1E,F). When back-crossed with the WT, all the plants of F_1_ generation resembled the male fertility of the WT (Figure S1). Moreover, in the F_2_ progeny, the plants with normal fertility and male sterility displayed an approximate segregation ratio of 3:1 (Table S1). These observations indicated that *pl1* mutant exhibited a completely male-sterile phenotype, which is caused by a single recessive mutation.

### 2.2. Cloning of PL1

**Figure 2:**
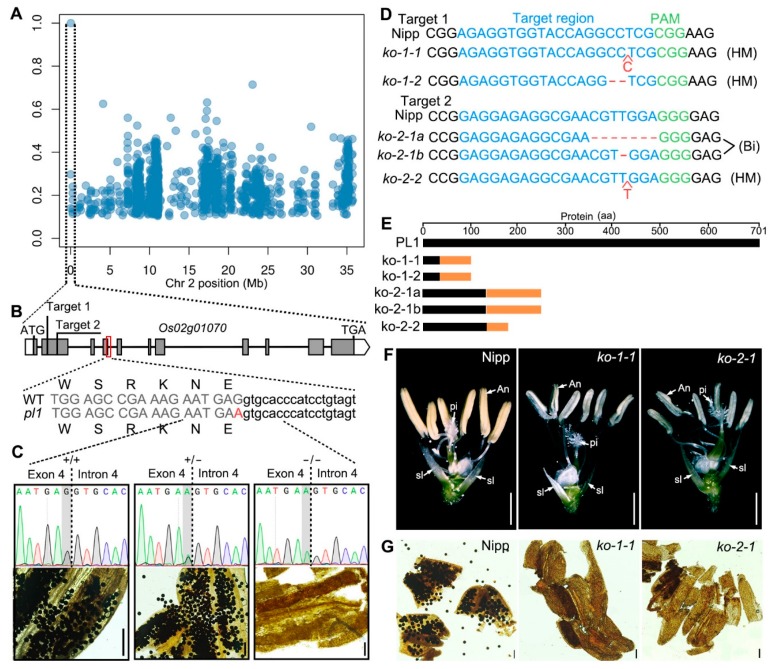
Cloning of *PL1* gene. (**A**) SNPs Plot on chromosome 2 (Chr 2) identified by MutMap analysis. (**B**) The gene structure of *PL1*. Genomic sequences around the mutated position of WT and *pl1* and two independent CRISPR/Cas9 target sites are shown. Grey boxes indicate exons. Intervening lines indicate introns. White boxes indicate untranslated regions. Parts of sequences of exon 4 and intron 4 are shown in upper case (grey) and lower case (black), respectively. The *pl1* mutation is highlighted with red color. (**C**) Genotypic and phenotypic identification of plants in F_2_ population. The mutated position is highlighted with a grey background. (**D**) Genomic sequencing of the CRISPR/Cas9 target regions in Nipponbare (Nipp) and *knock-out* (*ko*) lines. Bi, biallelic mutation. (**E**) Schematic diagrams of the amino acid changes of PL1 in *ko* lines. Unchanged and changed amino acids are shown in black and orange color, respectively. aa, amino acids. (**F**) Spikelet of Nipp and ko lines after removing of the lemma and palea. An, anther; pi, pistil; sl, sterile lemma. (**G**) I_2_ -KI staining of pollen grains of Nipp and *ko* lines at maturing stage. Bars = 100 μm in (**C**) and (**G**), and 1 mm in (**F**).

To unravel the casual mutation of *pl1*, we employed the MutMap method to analyze the single nucleotide polymorphisms (SNPs) positions of sterile individuals in the F_2_ progeny mentioned above [51]. The analysis of genomic resequencing results identified that only one SNP on chromosome 2 had a SNP index of 1 (Figure 2A and Figure S2), implying this SNP might be co-segregated with the mutant phenotype. Sequence analysis revealed that this point mutation (c. 777G > A) was located in the 3′ end of the fourth exon of *Os02g01070* but did not alter the encoded amino acid (Figure 2B), indicating this mutation is a silent mutation (p. E259E). To further verify the association between the mutation and the male-sterile phenotype of *pl1*, we analyzed the genotype and phenotype of F_2_ individuals. The results showed that, in F_2_ population, all the male-sterile plants carried the homozygous mutation, while the plants with normal fertility were WT or heterozygous (Figure 2C and Table S1). This result suggests that the mutation was co-segregated with the male-sterile phenotype of *pl1*, and that *Os02g01070* may be the causal gene.

To confirm that Os02g01070 is PL1, we designed two independent targets within the second exon of Os02g01070 (Figure 2B) and generated other allelic mutants (knocking out plants, ko plants) of pl1 using the CRISPR/Cas9 technology. In T_0_ plants, four mutants (three homozygous and one bi-allelic mutants in a background of japonica rice Nipponbare, Nipp) with five types of mutations were identified (Figure 2D). All these five mutations caused the production of premature and truncated proteins (Figure 2E and Figure S3A), indicating all mutants were effective loss-of-function mutants. Though these mutants displayed indistinguishable vegetative development and flower morphology compared with Nipp (Figure S3B,C), they had smaller and pale-yellow anthers (Figure 2F and Figure S3D). Moreover, the mature anthers of these mutants lacked pollen grains (Figure 2G), mimicking the phenotypes of pl1. We also back-crossed these mutants with Nipp and obtained several F_2_ populations. The genotypic and phenotypic association analysis of the segregated F_2_ populations found that the mutated genotypes were co-segregated with male sterility (Table S2). Together, these results confirmed that the mutation in Os02g01070 is responsible for the defective male-fertility in pl1. In another parallel study, other mutations in Os02g01070, which was named as Defective Pollen Wall 3 (DPW3), were also identified and consistently lead to the similar male-sterile phenotype [52].

### 2.3. Normal Splicing of PL1 Is Altered by pl1 Mutation

**Figure 3:**
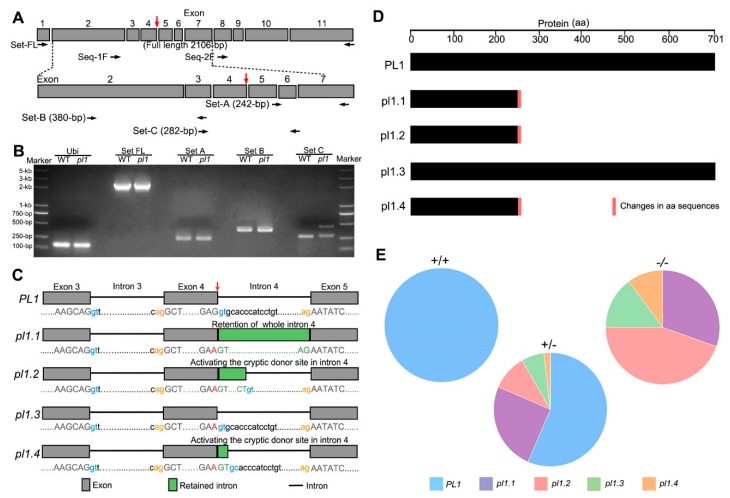
Identification of the *pl1* mutation associated missplicing. (**A**) Schematic diagram of positions of the primers used for RT-PCR. The *pl1* mutation site is indicated by red arrow. Grey boxes indicate exons. (**B**) RT-PCR analysis of *PL1* gene in the WT and *pl1*. The expected 242-bp and 380-bp products of the *PL1* with primer Set-A and Set-B, respectively, were detected in the WT and *pl1* plants, while additional larger products were observed in the *pl1* with primer Set-C. (**C**) Schematic diagrams of splicing patterns of the *pl1* mRNA species. *PL1* is the mRNA that is normally spliced in WT. *pl1.1* is the mRNA with retention of whole intron 4 in *pl1* mutant. *pl1.2* and *pl1.4* are mRNA spliced using the cryptic splice donor sites of intron 4 in *pl1* mutant. *pl1.3* is the normal spliced mRNA but harboring the *pl1* mutation. The splice donor sites are highlighted with blue color. The splice acceptor sites are highlighted with orange color. The *pl1* mutation site is indicated by red arrow, and the mutation is also highlighted with red color. Parts of sequences of exons and introns are shown in upper case and lower case, respectively. (**D**) Schematic diagrams of the amino acid changes of PL1 proteins translated from corresponding mRNA species. Unchanged and changed amino acids are shown in black and red color, respectively. aa, amino acids. (**E**) Comparisons of mRNA species ratios among plants with different genotype. The pie charts are used for visualizing the ratios.

Real-Time PCR (RT-PCR) analysis results showed that the level of full-length *PL1* cDNA detected in young spikelets of *pl1* seemed to be similar to that of the WT (Figure 3A,B), suggesting the *pl1* mutation may not affect *PL1* mRNA accumulation. We then purified the full-length RT-PCR products of the WT and *pl1* and designed two additional primers to sequence them (Figure 3A). The sequencing chromatogram resulted from Set-1F (a forward primer designed to mask the *pl1* mutation site, upper panel in Figure 3A) displayed the superimposed peaks after the c. 777G > A mutation in *pl1* (Supplementary Figure S4), suggesting a mixture of the full-length PCR products. We next designed three primer sets (lower panel in Figure 3A) and amplified the cDNA region of interest from the WT and *pl1* using RT-PCR analysis. We observed similar bands of the expected size in both the WT and *pl1* using primer set-A or set-B; however, we detected an additional large PCR product in the *pl1* using primer set-C (Figure 3B). These results suggest that the *pl1* mutation generated abnormal transcripts of the *PL1* gene. 

To characterize the detailed transcriptional changes caused by the *pl1* mutation, we cloned the full-length *PL1* CDS of the young spikelets from the WT and mutant. As shown in Figure 3C and Figure S5, a total of four different CDS variants, namely *pl1.1*, *pl1.2*, *pl1.3* and *pl1.4*, in *pl1* were found. Compared with the *PL1* CDS in WT, *pl1.1* CDS had an insertion of the whole fourth intron; *pl1.2* CDS and *pl1.4* CDS retained part of the fourth intron of 13 base-pairs and two base-pairs, respectively, indicating the activation of a cryptic splice donor site in the fourth intron. Interestingly, *pl1.3* CDS showed an intact splicing corresponded to the *PL1* CDS of WT but possessed the *pl1* mutation (c. 777G > A), which is a synonymous coding polymorphism (p. E259E). However, all the three alternative intron retentions shifted the open reading frame and yielded a premature stop codon (Figure 3D and Figure S6). Considering that the male-sterility of *pl1* mutant might be caused by the reduction of correctly processed PL1 protein, we calculated the corresponding colony numbers for each of the four transcripts by sequencing to quantify their relative amounts. The statistical results revealed that in *pl1* (*pl1*/*pl1*) young spikelets only ~15% (*pl1.3* type) of the *PL1* pre-mRNA was correctly processed, while the other three abnormal transcripts amounted to ~85% of the total (Figure 3E and Supplementary Table S3). Besides, we also investigated the splicing of *PL1* gene in the heterozygous plants (*PL1*/*pl1*) and found that the ratio of abnormally spliced *PL1* transcripts was ~37% (including *pl1.1*, *pl1.2* and *pl1.4*, Figure 3E and Table S3). Based on these results, we demonstrated that the *pl1* mutation is a leaky mutation that altered *PL1* splicing.

It was reported that a single point mutation was able to alter the pre-mRNA secondary structure and induce splicing errors [53,54]. The *pl1* mutation was at the end of the fourth exon of *PL1* gene and was adjacent to a splice donor site; we therefore hypothesized this mutation might affect pre-mRNA secondary structure. To test this, we carried out the RNA secondary structure prediction of *PL1* pre-mRNA without or with the *pl1* mutation. The results showed that, compared with the stem-loop structure with a three-base-pair stem and a 10-nucleotide loop around the mutation site in the WT *PL1* pre-mRNA, the mutated *PL1* pre-mRNA had a shorter stem with two-base-pair and a longer loop with 14-nucleotide (Figure S7). Additionally, in the mutated pre-mRNA, the minimum free energy (MFE) increased from –17.7 to –15.3 (Figure S7). These findings suggest that the *pl1* mutation may affect the stability of *PL1* pre-mRNA secondary structure, which in turn causes the abnormal pre-mRNA splicing. 

### 2.4. PL1 Encodes a Conserved Integrin-α FG-GAP Repeat-Containing Protein

**Figure 4:**
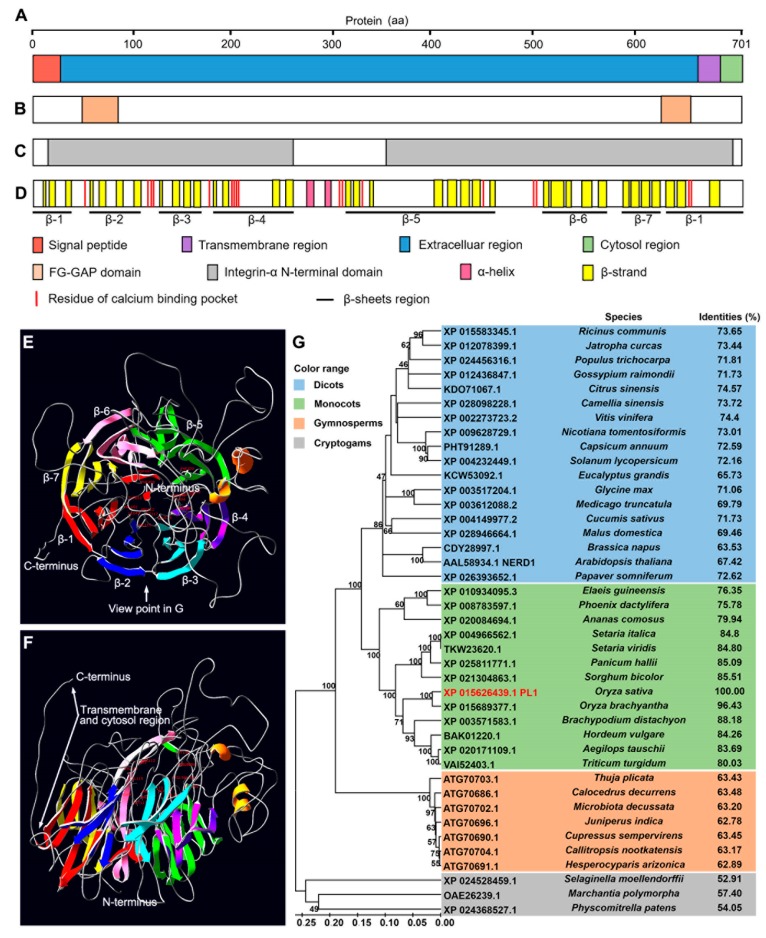
Protein sequence analysis of PL1. (**A**) Domains of PL1 determined by TMHMM 2.0. aa, amino acids. (**B**) FG-GAP repeat domains of PL1 determined by SMART. (**C**) Integrin α N-terminal domains of PL1 determined by InterProScan. (**D**) Seven-bladed β-propeller and residues of Ca2+-binding pocket predicted by RaptorX. (**E**) Ribbon diagrams of the model for PL1 predicted by RaptorX. (**F**) Side view of the model in E. Each β-sheets (from β-1 to β-7) is shown in a different color. The N-terminus, C-terminus and the transmembrane and cytosol region are indicated. (**G**) Phylogenetic tree of PL1 and its related homologs. The proteins are named according to their GenBank IDs (Table S4). The numbers at the nodes indicate the bootstrap values.

According to the annotation from Rice Genome Annotation Project (RGAP, http://rice.plantbiology.msu.edu/), *PL1* (*Os02g01070*) encodes an FG-GAP repeat-containing protein. An N-terminal signaling peptide followed by a large extracellular region, a single-pass transmembrane domain near the C-terminus and a short cytoplasmic tail was predicted in the protein using TMHMM 2.0 (http://www.cbs.dtu.dk/services/TMHMM/) (Figure 4A and Figure S8A). Furthermore, two FG-GAP repeat domains (Figure 4B and Figure S8B) and two integrin-α N-terminal domains were predicted using Simple Modular Architecture Research Tool (SMART, http://smart.embl-heidelberg.de/) and InterProScan (http://www.ebi.ac.uk/interpro/) (Figure 4C). The secondary and tertiary protein structure analysis showed these FG-GAP domains and integrin-α N-terminal domains were predicted to fold into seven β-sheets (Figure 4D and Figure S8B). These β-sheets arranged pseudosymmetrically in a torus around an axis (Figure 4E,F), which contains several putative residues to forming Ca^2+^-binding pocket (Figure 4D and Figure S8B). In addition, the structure of PL1 is similar to *Homo sapiens* (human) integrin α4-subunit [55] (Figure S8C).

To gain information about the evolutionary role of PL1, we searched its closest relatives using Basic Local Alignment Search Tool (BLAST) of National Center for Biotechnology Information (NCBI, https://blast.ncbi.nlm.nih.gov/Blast.cgi). Forty-one orthologues of PL1 were retrieved from different land plants species (Table S4). Subsequently, we constructed a phylogenetic tree using the neighbor-joining method. These proteins were grouped into four clades that belong to dicots, monocots, gymnosperms and cryptogams (Figure 4G). Protein sequence alignment analysis showed that PL1 shared high identities with the homologs identified, not only from primarily monocots and dicots but also from distantly related gymnosperms and cryptogams (Figure 4G and Figure S9 and Table S4). Protein sequence alignment and phylogenetic analysis also showed that NEW ENHANCER of ROOT DWARFISM 1 (NERD1), an integrin-α N-terminal domain containing protein required for *Arabidopsis* reproductive development [56,57], is an orthologue of PL1 (Figure 4G and Figure S9), suggesting a functional conservation between these two proteins. Overall, these results suggested that PL1 is a member of a highly conserved protein family in terrestrial plants.

### 2.5. Expression Analysis of PL1

**Figure 5:**
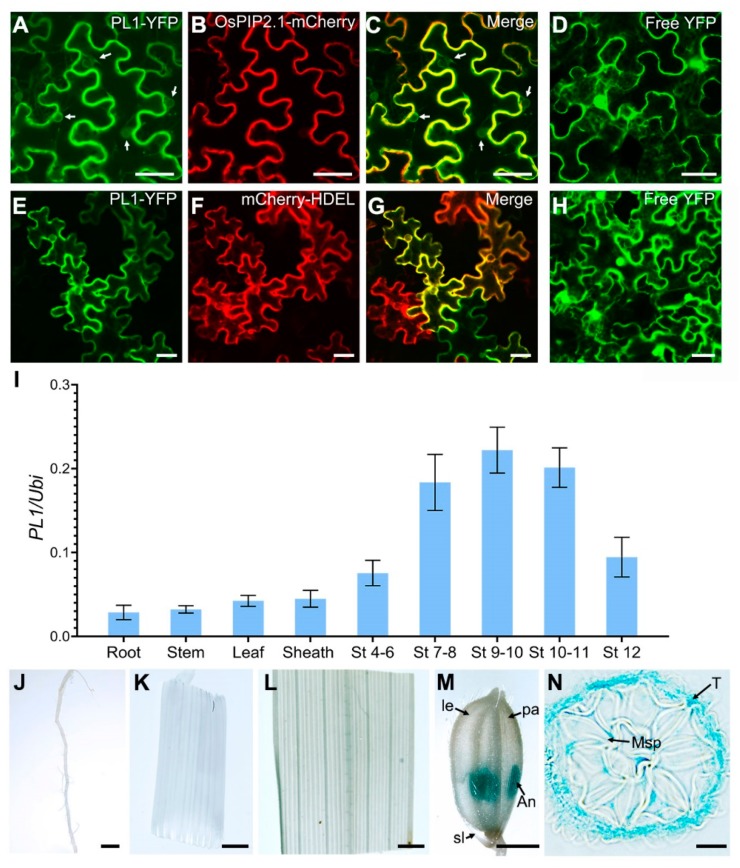
Expression analysis of *PL1*. (**A–H**) Subcellular localization analysis of PL1-YFP fusion protein in tobacco leaves. The co-expression of PL1-YFP and PM marker (OsPIP2.1-mCherry) is shown in (**A–D**). The co-expression of PL1-YFP and ER marker (mCherry-HDEL) is shown in (**E–G**). The free YFP that is used as a control is shown in (**D**) and (**G**). The arrows in (**A**) and (**C**) indicate the ER-like structures. (**I**) Expression pattern of *PL1*. The expression levels were examined by qRT-PCR. RNA was extracted from root, stem, leaf, sheath and spikelets with anthers at different developmental stages (St). Data are shown as means ± SD (n = 3). (**J–M**) GUS staining of the PL1pro::GUS transgenic plant. GUS activity in root (**J**), stem (**K**), leaf (**L**), spikelet with anthers at stage 10 (**M**). (**N**) Cross-section of anthers from (**M**). Bars = 10 µm in (**A–H**), 2 mm in (**J–M**) and 25 µm in (**N**).

The PL1 protein contains a transmembrane domain, suggesting this protein may be present on membrane. To validate this, we determined the subcellular localization of PL1 by transient co-expression of PL1-YFP fusion protein and a PM marker (OsPIP2.1-mCherry) in *Nicotiana benthamiana* (tobacco) leaf epidermal cells. Results showed that the fluorescent signals of PL1-YFP partially overlapped with the PM marker (Figure 5A–C). Besides, we observed that part of the YFP signals appeared on ring-like structures similar to ER (Figure 5A,C). To verify this speculation, we then examined the tobacco leaf epidermal cells co-expressing of PL1-YFP with an ER marker (mCherry-HDEL). Confocal microscopy revealed that the YFP signal co-localized with the ER marker (Figure 5E–G), while the free YFP (control) was expressed in the whole cell (Figure 5D,H). These results indicate that PL1 is primarily localized to PM and ER.

The loss-of-function of *PL1* resulted in a male-sterile phenotype, suggesting that *PL1* should be expressed in developing anthers. We used quantitative RT-PCR (qRT-PCR) to analyze the expression profiles of *PL1* in various tissues of Nipp plants. The results revealed that *PL1* transcripts were highly accumulated in developing spikelets with anthers from stages 4 to 12 and were particularly reached the maximum amount in spikelets with anthers at stages 9 and 10 (Figure 5I), when the tapetum PCD and pollen wall formation began (Zhang and Wilson, 2009; Zhang et al., 2011). However, relatively lower expressions of PL1 were detected in root, stem, leaf and sheath (Figure 5I). To refine this expression pattern, we transformed a *PL1pro::GUS* construct into Nipp plants. In transgenic plants, negligible GUS activity was present in vegetative tissues (including root, stem and leaf), and hull (including lemma and palea) and sterile lemma of spikelet with anthers at stage 10 (Figure 5J–M), while the strong GUS signals were observed in the anthers (Figure 5M), which resembled the qRT-PCR data. Moreover, observations on transverse sections of the anthers in Figure 5M found a specific GUS expression in tapetal cell layer (Figure 5N). These results indicated that PL1 is mainly expressed in tapetal cells of developing anther.

### 2.6. Cytological Observations of pl1 Anthers

**Figure 6:**
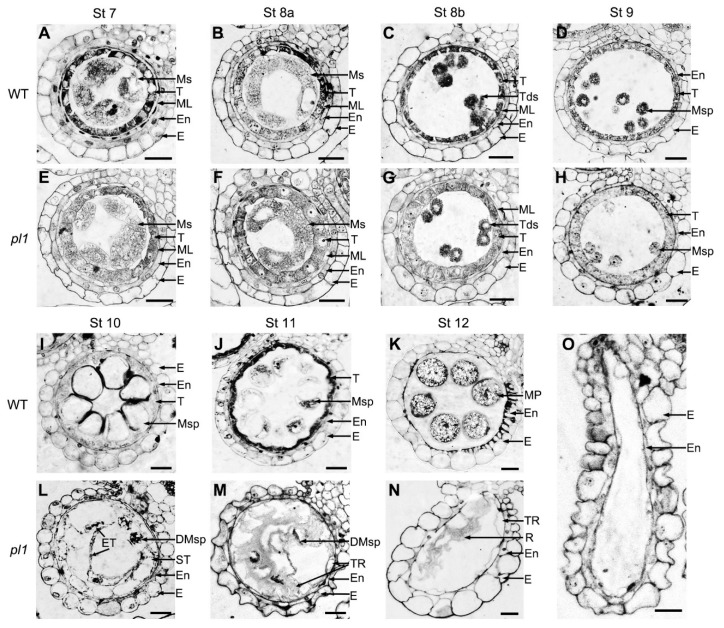
Cross-sections of anthers from stage (St) 7 to 12 in WT and *pl1*. (**A–D**) and (**I–K**) WT anthers from St 7 to 12. (**E–H**) and (**L–N**) *pl1* anthers from St 7 to 12. (**O**) *pl1* anther with an empty locule at stage 12. DMsp, degrading microspores; E, epidermis; En, endothecium; ET, ectopic tapetal cells; ML, middle layer; MP, mature pollen; Ms, microsporocyte; Msp, microspores; R, remnants; ST, swollen tapetal cells; T, tapetum; Tds, tetrads; TR, tapetal remnants. Bars = 25 μm.

To understand the dedicated role of *PL1* in male fertility, we compared transverse sections of anthers from both WT and *pl1* mutant based on their developmental stages [9,10]. From stage 7 to stage 9 (Figure 6A–H), the PMCs meiotic processes of WT and *pl1* anthers were indistinguishable, and their microspore were normally released from the tetrads. During these stages, the WT tapetum developed as expected and started to condense (Figure 6A–D), but the tapetal cell layers of *pl1* showed a slightly swollen morphology (Figure 6E–H). At stage 10, the WT microspores enlarged and vacuolated, meanwhile, its tapetum underwent degradation into thin and hill-like layer (Figure 6I). On the contrary, in *pl1* anthers, tapetal cells seemed to be ectopically hypertrophic with less cytoplasm contents, and the microspores had abnormal shape with the tendency to degenerate and without vacuolation (Figure 6L). During stage 11, the WT microspores went through two steps of mitosis, and the tapetum degraded further (Figure 6J). However, the *pl1* microspores were shriveled and crushed, while the tapetum rapidly degenerated and part of the remnants invaded into the locular space (Figure 6M). At stage 12, the WT tapetal cell layer disappeared completely, and the microspores developed into mature pollen grains filled with inclusions (Figure 6K). By contrast, the *pl1* anthers had locules containing remnants of degraded tapetum and microspores (Figure 6O) or empty locules (Figure 6N). 

**Figure 7:**
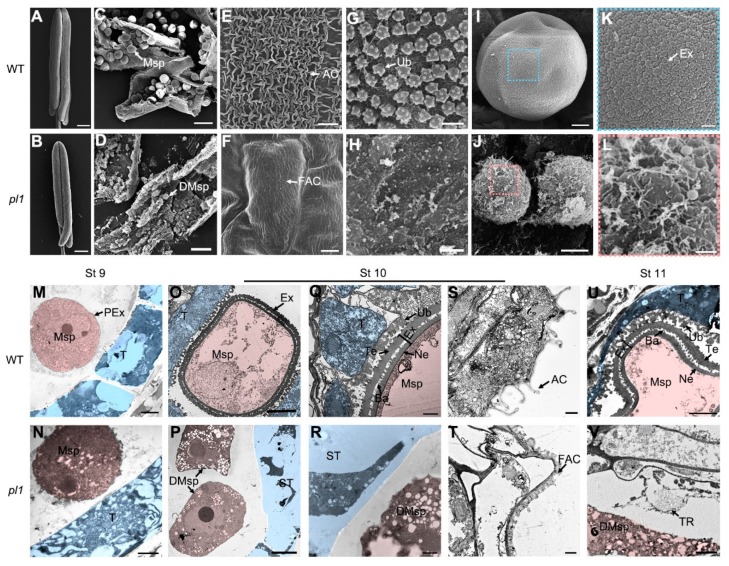
SEM and TEM analysis of the anthers in WT and *pl1*. (**A–L**) SEM observations of the surfaces of anther and microspores at stage 10 in WT and *pl1*. M-V TEM analysis of the anther sections in WT and *pl1* from stages (St) 9 to 11. Microspores are false colored in orange. Tapetum is false colored in blue. A and B The appearance of entire anther of WT (**A**) and *pl1* (**B**). (**C**,**I**) Microspores in WT anthers. (**D,J**) Microspores in *pl1* anthers. (**E,F**) The outer surface of the epidermis of WT (**E**) and *pl1* (**F**) anthers. (**G,H**) The inner surface of tapetum of WT (**G)** and *pl1* (**H**) anthers. (**K,L**) The enlarged view of the microspore surface of WT (**K**) and *pl1* (**L**). (**M,N**) Tapetum and microspore at stage 9 in WT (**M**) and *pl1* (**N**). (**O,P**) Tapetum and microspore at stage 10 in WT (**O**) and *pl1* (**P**). (**Q,R**) Tapetal cells and pollen exine at stage 10 in WT (**Q**) and *pl1* (**R**). (**S,T**) The outer region of anther epidermis at stage 10 in WT (**S**) and *pl1* (**T**). (**U,V**) Tapetal cells and pollen exine at stage 11 in WT (**U**) and *pl1* (**V**). AC, anther cuticle; Ba, bacula; DMsp, degrading microspores; Ex, exine; FAC, flat anther cuticle; Msp, microspores; Ne, nexine; PEx, primexine; ST, swollen tapetal cells; T, tapetum; Te, tectum; TR, tapetal remnants; Ub, Ubisch bodies. Bars = 200 μm in (**A**) and (**B**), 50 μm in (**C**) and (**D**), 5 μm in (**E**), (**F**), (**I**), (**J**) and (**M**–**P**), and 1 μm in (**G**), (**H**), (**K**), (**L**) and (**Q**–**V**).

To gain a more detailed understanding of the anther development process that *PL1* might affect, electron microscopic analyses were further performed. We examined anthers at stage 10 by scanning electron microscope (SEM) and observed a smaller size of *pl1* anthers that had less and deformed microspores (Figure 7B,D) compared with those of the WT (Figure 7A,C). Moreover, in WT anthers, the spaghetti-like cuticle (Figure 7E) and granular Ubisch bodies (Figure 7G) were apparent on the epidermal surface of anther wall and inner locule surface of tapetum, respectively. In contrast, outer surface of *pl1* anther had flat and smooth nanoridges (Figure 7F), and its inner surface lacked Ubisch bodies (Figure 7H). Besides, unlike the round shape microspores with elaborate exine pattern in the WT anther (Figure 7I,K), the exine development of *pl1* microspores at this stage appeared to be arrested and be intended to collapse (Figure 7J,L), which is consistent with the light microscopic observations.

We also utilized transmission electron microscope (TEM) to further characterize the developing defects in *pl1* anthers. At stage 9, no discernable differences of tapetum development between WT and *pl1* anthers were observed (Figure 7M,N). Microspores in WT anthers were released form tetrads and displayed a thin layer of primexine (Figure 7M), while no obvious primexine structure appeared on the PM surface of released *pl1* microspores (Figure 7N). At stage 10, the WT anthers had a condensed tapetal cell layer with conical Ubisch bodies covered on and highly vacuolated microspores with double-layered exine (contained tectum, bacula and nexine; Figure 7O,Q). While at this stage, in *pl1* anthers, abnormally swollen tapetal cells with low electron-dense cell wall and fragmented cytoplasm were observed, and the formation of Ubisch bodies was devoid (Figure 7P,R). In parallel, the *pl1* microspores did not have any exine-like structure and began to collapse (Figure 7P,R). Additionally, the surface of the WT anther exhibited well-developed cuticle (Figure 7S), whereas that was flat and short in *pl1* (Figure 7T). At stage 11, the tapetum was more condensed, and the microspores became falcate in WT anthers (Figure 7U). By contrast, in *pl1*, the tapetal cells and microspores had degenerated circumstantially, leaving the remnants in the locule (Figure 7V). Together, these observations indicated that *PL1* is indispensable for anther cuticle, tapetum and pollen development.

### 2.7. The pl1 Mutation Altered the Expression of Genes Related to Anther and Pollen Development

**Figure 8:**
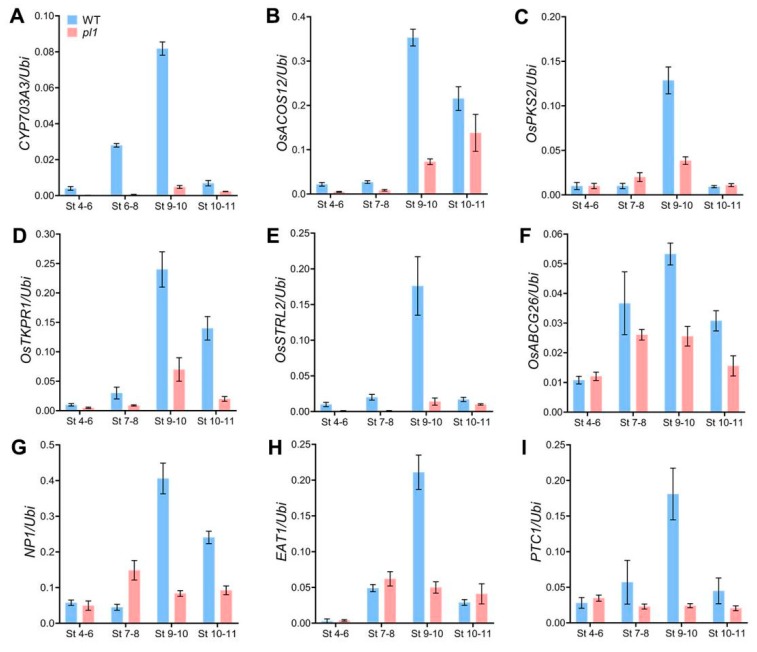
Expression profiles of genes related to anther and pollen development in WT and *pl1*. Expression levels of *CYP703A3* (**A**), *OsACOS12* (**B**), *OsPKS2* (**C**), *OsTKPR1* (**D**), *OsSTRL2* (**E**), *OsABCG26* (**F**), *NP1* (**G**), *EAT1* (**H**) and *PTC1* (**I**) in spikelets with anthers at different stages (St) were analyzed using qRT-PCR. Data are shown as means ± SD (n = 3).

To explore the role of *PL1* in anther cuticle, tapetum and pollen development, we analyzed expression profiles of several known genes, which were critical for anther and pollen development, in WT and *pl1* spikelets with anthers from stages 4 to 11 by qRT-PCR. We first monitored *CYP703A3* [24], *OsACOS12* [26,27], *OsPKS2* [28,29] and *OsTKPR1* [32], which are required for pollen exine formation and lipid metabolism in tapetal cells. Our analysis showed that the expression levels of these genes were decreased in *pl1* (Figure 8A–D). Next, we examined rice *Strictosidine synthase-like 2* (*OsSTRL2*) because it plays important role in pollen exine formation [58], the results showed a dramatically down-regulated expression of *OsSTRL2* (Figure 8E). These results suggested a possibly defective biosynthesis of pollen exine components in the mutant. 

We subsequently compared the expression of genes involved in the anther development, including anther cuticle formation, Ubisch bodies pattering, and tapetal PCD. Transcript levels of *OsABCG26*, which transports wax and cutin monomers from tapetal cells to anther surface for anther cuticle development [34,59], was not significantly changed at early stages but reduced during stages 7 to 11 (Figure 8F). *No Pollen 1* (*NP1*) is also crucial for formation of anther cuticle and patterning of both Ubisch bodies and pollen exine [60,61]. An increase in the expression level of *NP1* was observed in WT from stages 7 to 10, whereas it was decreased during these stages in the mutant (Figure 8G). In addition, *EAT1* and *PTC1* are related to tapetal PCD [19,23]. The expression pattern of *EAT1* in *pl1* was similar to that of the WT from stages 4 to 8, but at stages 9 and 10, the transcript levels of *EAT1* significantly decreased (Figure 8H). Similarly, a down-regulation of *PTC1* expression was also detected from stages 7 to 11 in *pl1*, compared with that of the WT (Figure 8I). The reduced expressions of these genes were consistent with the defective anther development in *pl1*.

## 3. Discussion

### 3.1. The pl1 Mutation Affects Splice Donor Site Selection

In eukaryotes, gene expression starts from the generation of pre-mRNA, a copy of the genomic DNA containing intronic regions [62]. Next, the pre-mRNA is modified to become the mature mRNA for translation. This modification, called RNA splicing, is a process that removes introns and joins exons [63]. RNA splicing is tightly regulated by the complexes known as spliceosomes, which are comprised of multiple small nuclear RNA (snRNA) and small nuclear ribonucleoproteins (snRNPs) [64,65]. For accurate splicing of intron, the spliceosomes need to recognize the intrinsic regulatory sequences: the splice donor sites (at 5′ end of the introns), splice acceptor sites (at 3′ end of intron) and exonic splicing enhancers [40,66]. These sequences are base-paired with the different snRNA of spliceosomes and play variant roles in RNA splicing [39,64]. Abnormal splicing, such as exonic skipping and intronic retention, are most commonly caused by mutations in splice donor or acceptor sites of intron [67,68,69]. In the present study, we revealed that a novel exonic mutation resulted in missplicing and male sterility in rice.

In RNA splicing processes, the recognition of splice donor sites requires U1 snRNA of spliceosome base-paring with one nucleotide upstream and six nucleotides downstream around exon-intron boundaries [64,66,69,70,71]. In silico analysis shows that the *pl1* mutation did not induce an amino-acid change of *PL1*; however, this mutation is positioned at the last base of the fourth exon, which is in close proximity to the splice donor site of the fourth intron (Figure 2B). Via combined analysis of RT-PCR and sequencing, we revealed that the *pl1* mutation generated transcript aberrations of *PL1*. All the aberrant mRNA species had retained the original splice donor site of the fourth intron (Figure 3 and Figure S5), suggesting that the *pl1* mutation may influence the base-paring and modification of adjacent splice donor site of the fourth intron in spliceosome. Besides, previous studies suggested the structure of hairpins in pre-mRNA is also important for its stability and splice site selection [72,73,74]. Our secondary structure prediction of pre-mRNA indicated the *pl1* mutation expanded the adjacent loop and shortened the stem, respectively, and also increased the MFE (Figure S7), suggesting a reduced stability of *pl1* pre-mRNA. Based on these results, we propose that the *pl1* mutation would shift the base-pairing with U1 snRNA and influence the stability of pre-mRNA secondary structure, thereby affecting the normal recognition of the adjacent splice donor site.

The conventional introns have well-conserved splice donor sites with 5′-GT boundaries [75]. Inactivation of the normal splice donor sites would result in the activation of cryptic splice sites in the adjacent exon and intron [76]. It is worth noting that, in this study, parts of these aberrant mRNA species (*pl1.2* and *pl1.4*) were caused by activating the cryptic donor sites in the fourth intron (Figure 3C). Among them, we found a non-canonical 5′-GC type, by which the 5′ border of the fourth intron in *pl1.4* was demarcated (Figure 3C and Figure S5). This suggests that the 5′-GC boundaries in the splice donor sites may also ensure the constitutive pre-mRNA splicing in rice, similar to results in other eukaryote species [77,78,79].

### 3.2. Normal Rice Male Development Requires a Certain Amount of Functional PL1 Protein

Disruptions of *PL1*′s function by two independent CRISPR/Cas9 targets led to male-sterility (Figure 2D–G), suggesting the functional *PL1* protein is crucial for normal male development in rice. The *pl1* mutation did not result in any amino acid substitution (Figure 2B), inferring that the normal splicing of *PL1* pre-mRNA carrying this mutation also generated functional protein. Indeed, although rare, normally spliced transcripts (*pl1.3*) were detected in both heterozygous and homozygous mutated plants (Figure 3E and Table S3). It should be emphasized that the aberrant mRNA species (*pl1.1*, *pl*1.2 and *pl1.4*), which were predicted to produce non-functional truncated PL1 proteins, were not found in WT plants but exhibited different ratios between the heterozygous and homozygous mutated plants. Correspondingly, in *pl1* (homozygous mutant), normally spliced transcripts that accounted for only ~15% of all transcripts, were much fewer than ~63% in heterozygotes (Figure 3E and Table S3). Consistently, only homozygous mutated plants harbored male sterility while the heterozygous ones were normally fertile (Figure 2C and Figure S1 and Table S1). It is therefore conceivable that, though the amount of functional *PL1* protein in heterozygous plants is sufficient for maintaining the normal male-fertility, it is not enough in *pl1*. Thus, we speculate that a certain amount of functional PL1 protein may be required for rice male development; however, further efforts are needed to determine its specific threshold value for fertility alteration, which might have applications in manual regulation of rice male fertility.

### 3.3. Role of PL1 in Anther and Pollen Development

Similar to *defective pollen wall 3* (*dpw3*), another allelic mutant of *pl1* (in *japonica* background) reported very recently [52], our *pl1* mutant (in *indica* background) exhibited slightly swollen tapetum and abnormal primexine matrix formation at stage 9 (Figure 6H and Figure 7N), and necrosis-like tapetum cell death with less cytoplasmic contents, severely enlarged cell size and compromised cell wall integrity, as well as defective formation of Ubisch bodies and anther cuticle at later stages (Figure 6L–O and Figure 7P,R,T). However, *pl1* had some characteristics distinguished from *dpw3*. Firstly, at stage 10, *pl1* showed degeneration of microspores with no deposition of sporopollenin precursors on their surface (Figure 7P,R,T), while bead-like sporopollenin precursors gathers around the degrading microspores in *dpw3*. Secondly, in addition to hypertrophy, some of the tapetal cells in *pl1* ectopically invaded into the locular space and subsequently degraded into remnants from stages 10 to 12 (Figure 6L–N). By contrast, in *dpw3*, the tapetal cells with atypical degeneration and enlargement persisted through stage 10 and stage 11. These different cytological features may be due to the large background differences of these two mutants or the differential biochemical alterations of their independent mutations. Nevertheless, it is clear that *PL1* is indispensable for normal pollen exine formation, tapetum PCD and anther cuticle development in rice. 

Crisscross anther cuticles and two-layered pollen exine are the major protective structures for preventing the male reproductive system from attacks of pathogens and external environmental stresses [15]. Tapetum, the innermost layer of the anther, undergoes PCD in time, and supplies materials, such as sporopollenin precursors and cutin monomers, for the formation of pollen exine and anther cuticle [6]. Most enzymes catalyzing the lipid metabolism for bio-synthesis of sporopollenin precursors, and cutin monomers are preferentially expressed in the tapetal cells [6,8,15,31]. *PL1* is also highly expressed in the tapetum of developing anther (Figure 5I,M,N), suggesting a role of this gene in pollen exine and anther cuticle formation. Indeed, in *pl1*, the transcript levels of several key enzyme encoding genes involved in this process were down-regulated (Figure 8A–D). Consistently, loss-of-function of *PL1* decreased the levels of serval waxes components and dominant cutin monomers [52]. These data raise a possibility that *PL1* might participate in male development by affecting the metabolic pathway of anther cuticle and pollen exine. Besides, the *pl1* mutant also exhibited abnormal tapetum PCD (Figure 6H–N) and the reduced expression of genes related to tapetal development (Figure 8H,I). Because these genes have been known as transcription factors to activate the transcription of key genes involved in the metabolic pathways of sporopollenin precursors and cutin monomers in tapetal cells [5,8,23], we therefore cannot rule out the possibility that *PL1* may be linked to the development of the tapetal cell layer, thereby affecting the supply of the anther cuticle and pollen exine components. 

*PL1* putatively encodes an integrin-α FG-GAP repeat-containing protein with a seven-bladed β-propeller domain (Figure 4A–C), which shares a highly structural similarity with the human integrin α4-subunit [55] (Supplementary Figure S9C), suggesting that PL1 may resemble the function of integrin-like proteins. The FG-GAP domain of integrin α4-subunit has been predicted to bind Ca^2+^ [55]. Particularly noteworthy is the fact that rice Defective in Exine Formation 1 (OsDEX1) has the Ca2+-binding activity and also contains two FG-GAP repeat domains like PL1 [80]. Mutation of *OsDEX1* also causes defective tapetal cell degradation and pollen formation [80], which is similar to that of *pl1*, suggesting that PL1 may have a potential function in modulating the Ca^2+^ homeostasis during anther and pollen development. Additionally, phylogenetic analysis showed that PL1 has a high identity with *Arabidopsis* NERD1 (~67% identity, Figure 4G). Importantly, the *nerd1-2* mutant was also displayed in the phenotype of defective pollen development, suggesting a possible functional conservation of PL1 and NERD1 in male development between rice and *Arabidopsis*.

Male sterility is widely used to avoid laborious emasculation in rice hybrid seeds production [81,82]. With the advance of technology, a new male sterility system for crops hybrid breeding, namely Seed Production Technology (SPT), was developed. SPT uses recessive male-sterile mutant to generate a maintainer line that has capability to reproduce non-transgenic male-sterile lines. This technology has been applied in maize and rice successfully [60,83]. The *pl1* mutation identified in this work is recessive (Figure 2), and the homozygous *pl1* mutant had a complete male-sterile phenotype (Figure 1), suggesting a potential application of *pl1* in rice hybrid breeding with SPT.

## 4. Materials and Methods 

### 4.1. Plant Materials and Growth Conditions

The *pl1* mutant was identified from the progenies of *indica* rice variety 9311 after treating with EMS [51]. As the pollen acceptor, *pl1* was back-crossed with the WT to generate F_1_ and F_2_ populations for genetic analysis and gene mapping. Other additional mutant alleles of *PL1* (*ko* plants) were created by CRISPR/Cas9 genome editing tool. The CRISPR/Cas9 plasmids carrying specific target oligos (Table S5) were constructed and transformed into *japonica* rice variety Nipp in accordance with the methods described previously [84]. All the rice plants materials used in this study were grown in the paddy field of Lingshui, Hainan Province or in Wenjiang, Sichuan Province, and were managed following commercial rice production practices.

### 4.2. Phenotypic and Microscopic Observations

The whole plant and reproductive organs were photographed with a EOS 1200D digital camera (Canon, Tokyo, Japan). Anthers at the heading stage that contain mature pollen grains were squeezed, and were then stained with 1% I_2_-KI solution and observed using a Axio Lab. A1 microscope (Zeiss, Baden-Wurttemberg, Germany). Spikelets with anthers at different developing stages were classified, and were fixed with Carnoy’s fixative or 2.5% glutaraldehyde fixative according to the previous description [84]. The subsequent dehydration, semi-thin sections analysis, SEM and TEM analysis of the anther samples were performed as described previously [30].

### 4.3. Mapping the pl1 Mutation

DNA from 50 male-sterile individuals characterized from F_2_ population was extracted and bulk-sequenced using Plant Genomic DNA Kit (Tiangen Biotech, Beijing, China) and Illumina sequencing platform (Novogene, Beijing, China), respectively. The sequence data were further subjected to computational analysis for detecting and annotating SNPs as described previously [51]. Co-segregation analysis of the candidate mutation in F_2_ population and the target site mutations in ko plants were carried out by PCR and sequencing with corresponding primer sets (Table S5) as described previously [30].

### 4.4. Pre-mRNA Secondary Structure Prediction

For prediction of Pre-mRNA secondary structure, the 101 nucleotides (including 50 nucleotides upstream and downstream of the mutation site) sequences with or without *pl1* mutation were submitted to the Mfold web server (http://unafold.rna.albany.edu/?q=mfold/download-mfold), and the resultant structures were reached by following the guidance described by Zuker [85].

### 4.5. RNA Extraction and RT-PCR Analysis

Total RNA was extracted from various rice tissues, including root, stem, leaf, sheath and spikelets with anthers at different developing stages, using Plant Total RNA Isolation Kit (Sangon Biotech, Shanghai, China) as described by the manufacturer. The first strand cDNA was synthesized using NovoScript^®^ Reverse Transcriptase (Novoprotein, Shanghai, China) as described by the manufacturer. 

RT-PCR analysis were performed in a regular 28 cycles of three steps PCR program using Biometra TOne thermal cycler (Analytik-Jena, Jena, Germany) with the corresponding primer sets (Table S1) and 2×TSINGKE Master Mix (Tsingke, Beijing, China). The products amplified by RT-PCR with primer Set-FL (Table S5) were further subcloned using 5min TA/Blunt-Zero Cloning Kit (Vazyme, Nanjing, China). More than 200 clones of each plant with different genotype were sequenced for calculating the ratio of each transcript variant. qRT-PCR were performed using qTOWER3G machine (Analytik-Jena, Jena, Germany) with the corresponding primer sets (Table S5) and AceQ Universal SYBR qPCR Master Mix (Vazyme, Nanjing, China). The expression level of *Ubiquitin* (*Os03g13170*) was used as the internal control for normalization of mRNA expression ratio.

### 4.6. Histochemical GUS Assay

A 2.6-kb upstream region of the *PL1* gene was amplified with primer set GUS-*PL1* (Table S1) and was then inserted into PHB-GUS vector to generate the *PL1*pro::GUS construct. This construct was further introduced into Nipp by Agrobacterium tumefaciens-mediated transformation [86]. Histochemical GUS staining of different rice tissues and section analyses of stained anthers were performed as described previously [58].

### 4.7. Subcellular Localization Analysis of PL1

To determine the subcellular localization of PL1, a full-length cDNA lacking a stop codon of *PL1* was amplified with primer set YFP-PL1 (Table S1) and was further inserted into PHB-YFP vector for generating the *2×35Spro::PL1-YFP* construct, in which the YFP was directly fused to the C-terminus of PL1. For co-localization of PL1-YFP with organelle markers, a rice PM aquaporin (OsPIP2.1) was fused to the N-terminus of mCherry to obtain the PM maker [87], and an HDEL retention signal was fused to the C-terminus of mCherry to obtain the ER marker [88]. The resulting constructs were transiently co-expressed in tobacco leaf epidermal cells following the method described previously [89]. The florescent signals were visualized using a A1 confocal scanning microscope (Nikon, Tokyo, Japan) at 48 h after infiltration.

### 4.8. Protein Sequence Analysis of PL1

For determination the domains in PL1, the full-length amino acid sequence of this protein, which was obtained from RGAP (http://rice.plantbiology.msu.edu/), was submitted to TMHMM 2.0 tool (http://www.cbs.dtu.dk/services/TMHMM/) in the Expert Protein Analysis System, SMART (http://smart.embl-heidelberg.de/), and InterProScan (http://www.ebi.ac.uk/interpro/) were performed with default settings. For secondary and tertiary protein structure prediction of PL1, the full-length amino acid sequence of this protein was submitted to an online threading program RaptorX (http://raptorx.uchicago.edu/), and the prediction were processed with default settings. The protein templates used to derive the resultant model of PL1 were listed in Supplementary Table S6. For phylogenetic analysis of PL1, the full-length amino acid sequence of this protein, as the query, was submitted to BLAST (https://blast.ncbi.nlm.nih.gov/Blast.cgi) of NCBI. Forty-one orthologues of PL1 from different species were identified (Table S4). All these orthologues and PL1 were aligned using ClustalW of MEGA 5.0 [90], and the alignment result was then applied to construct a neighbor-joining phylogenetic tree using MEGA 5 with 1000 bootstrap replicates.

## 5. Conclusions

To conclude, in the present work, we reported that a silent mutation at the last base of exon 4 in *PL1* gene caused the missplicing and completely male-sterile phenotype in rice. Our findings highlight the crucial roles of integrin-α FG-GAP repeat-containing protein in male reproduction and the exonic influence on splice donor site selection. Further experimental determinations on the biochemical functions of PL1 in anther and pollen development will be interesting. 

## Supplementary Materials

Supplementary materials can be found at https://www.mdpi.com/1422-0067/21/6/2018/s1.
