# Supplementary material for: A Silent Exonic Mutation in a Rice Integrin-α FG-GAP Repeat-Containing Gene Causes Male-Sterility by Affecting mRNA Splicing
Source: Int J Mol Sci. 2020 Mar 16;21(6):2018. doi: 10.3390/ijms21062018 (PMC7139555; doi:10.3390/ijms21062018)
Supplement: Supplementary file 1 [file ijms-21-02018-s001.pdf]

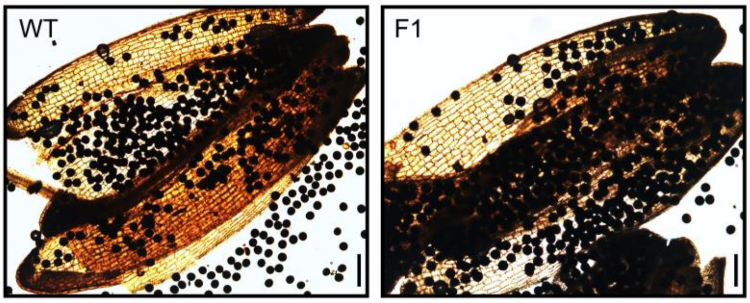

Figure S1. I2 -KI staining of pollen grains at mature stage in WT and F1 plants. Bars=100 μm.

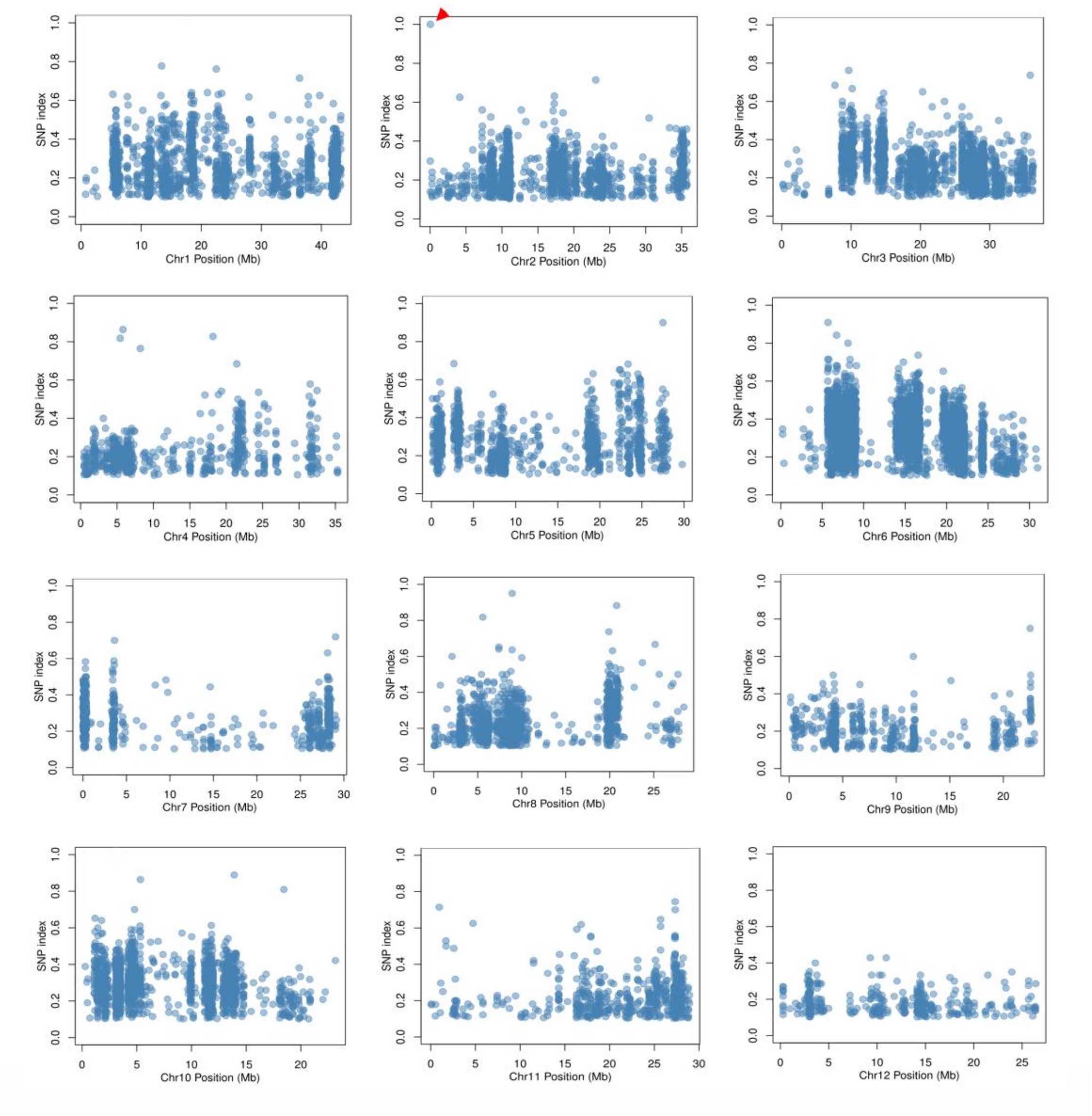

Figure S2. Distributions of SNP index along chromosomes. The candidate pl1 mutation on chromosome 2 is indicated by a red arrow.

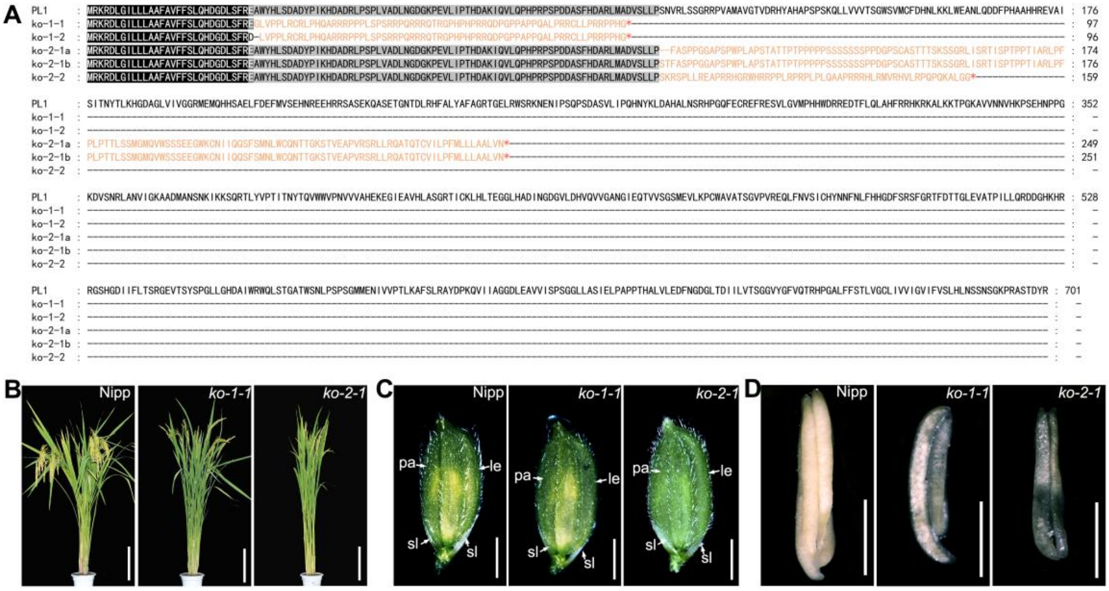

Figure S3. Protein sequences and phenotypic analysis of *knock-out* (*ko*) lines. (**A**) Protein sequences alignment of PL1 in ko lines. The sequences were displayed with BOXSHADE. The changed amino acids are highlighted by orange color. The red asterisks indicate the stop codons. (**B**) Nipponbare (Nipp) and *ko* plants at grain-filling stage. (**C**) Nipp and ko spikelet at heading stage. (**D**) Nipp and ko anther at heading stage. Bars=20 cm in (**B**), 2 mm in (**C**) and 1 mm in (**D**).

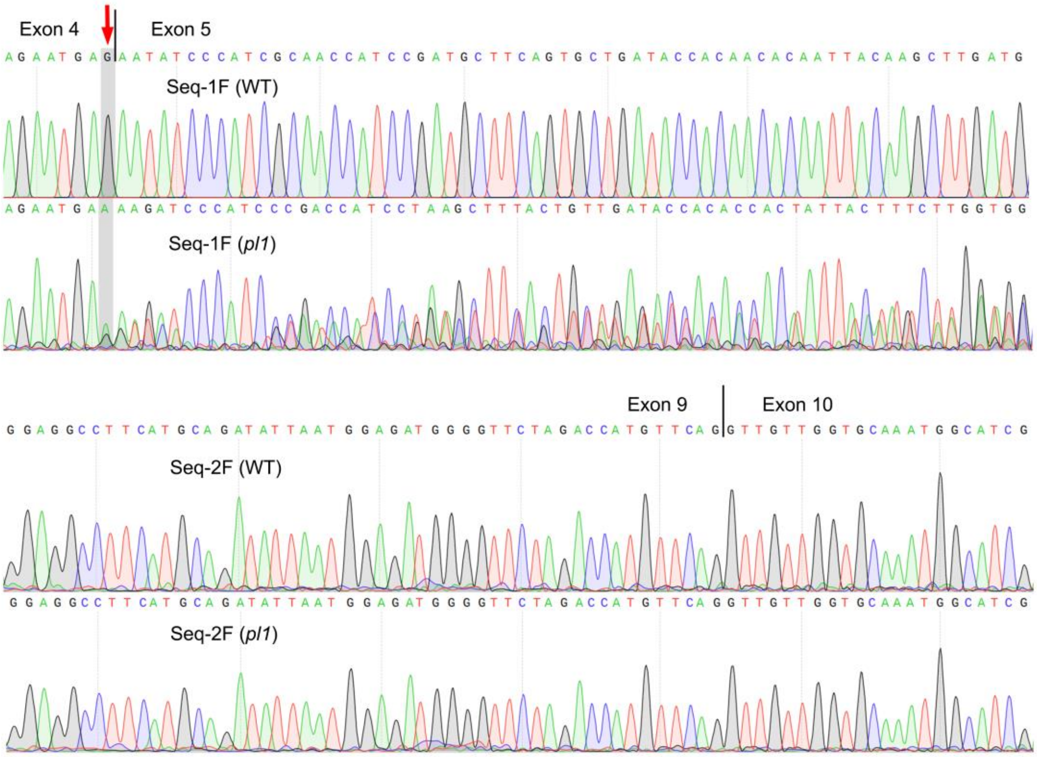

Figure S4. Sequence analysis of the RT-PCR products resulted from primer Set-FL. Sequencing results of WT and pl1 using the primer Seq-1F are illustrated in upper panel. Sequencing results of WT and pl1 using the primer Seq-2F are illustrated in lower panel. The pl1 mutation site is highlighted with gray background indicated by a red arrow.

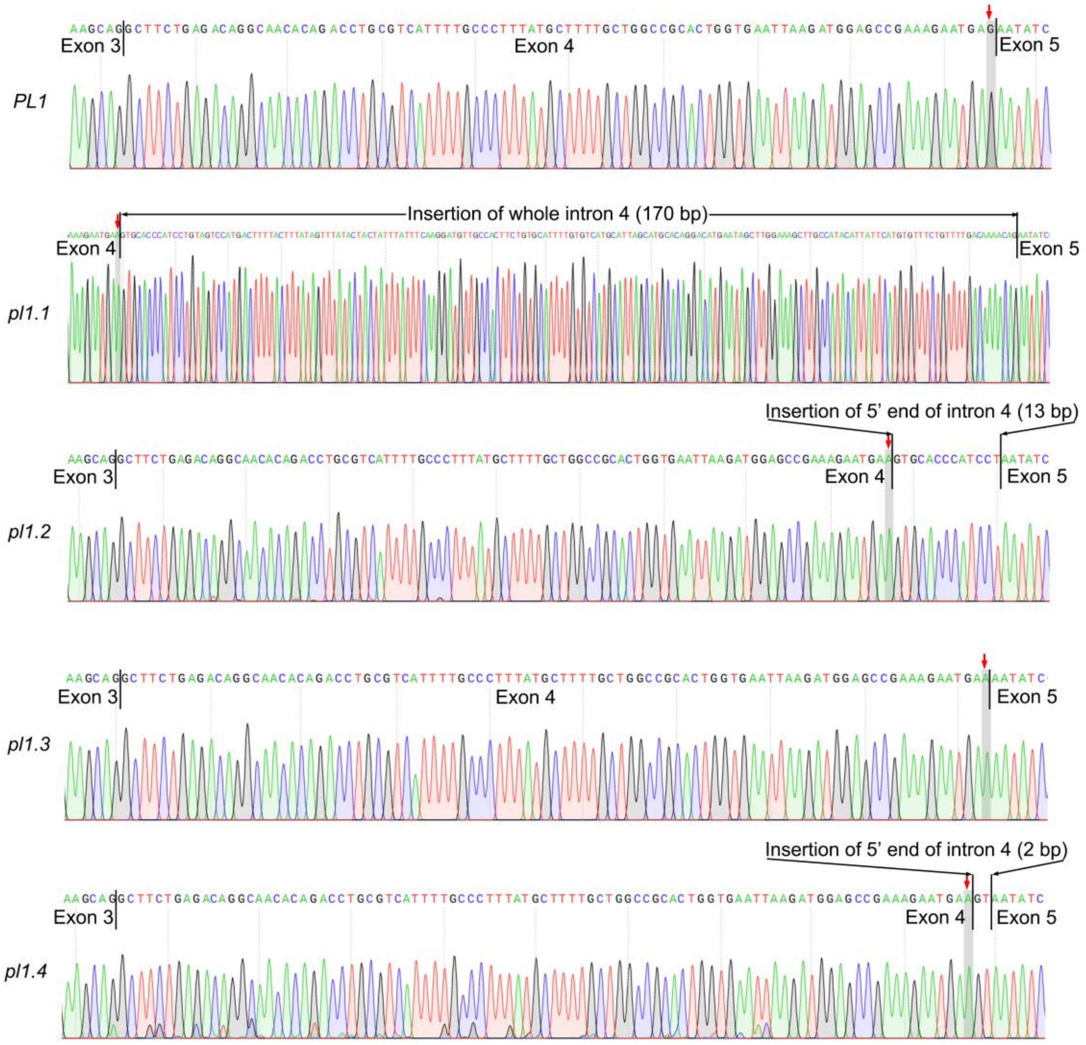

Figure S5. Identification of transcript aberrations. Representative sequencing results of WT and pl1 are illustrated. The pl1 mutation site is highlighted with gray background indicated by a red arrow.

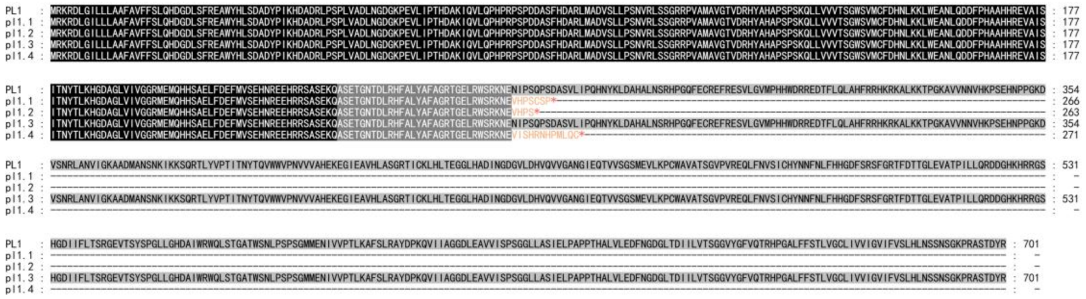

Figure S6. Amino acids alignment of proteins translated from each mRNA species. The sequences were displayed with BOXSHADE. The changed amino acids are highlighted by orange color. The red asterisks indicate the stop codons.

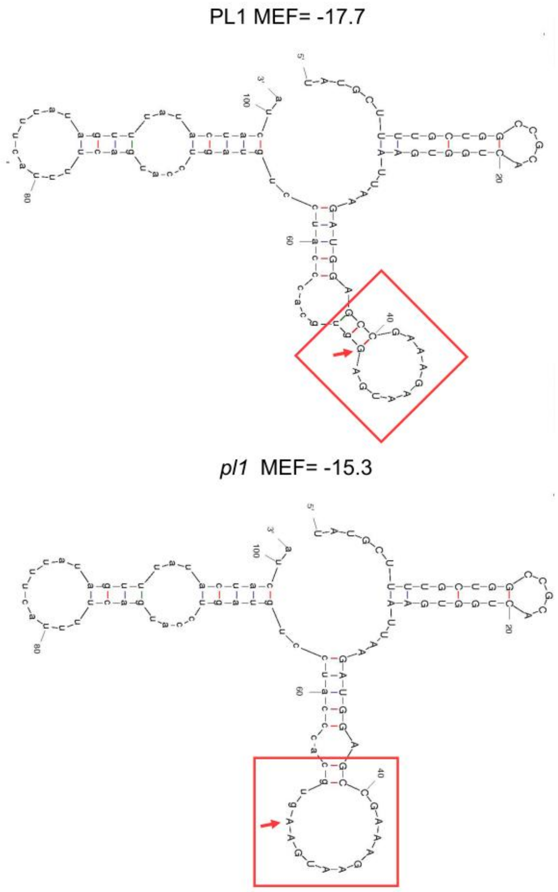

Figure S7. The *PL1* RNA secondary structure and MEF prediction of WT (upper) and *pl1* (lower) pre-mRNA. The *pl1* mutation site is indicated by a red arrow. The changes of stem-loop structure are highlighted by red frames.

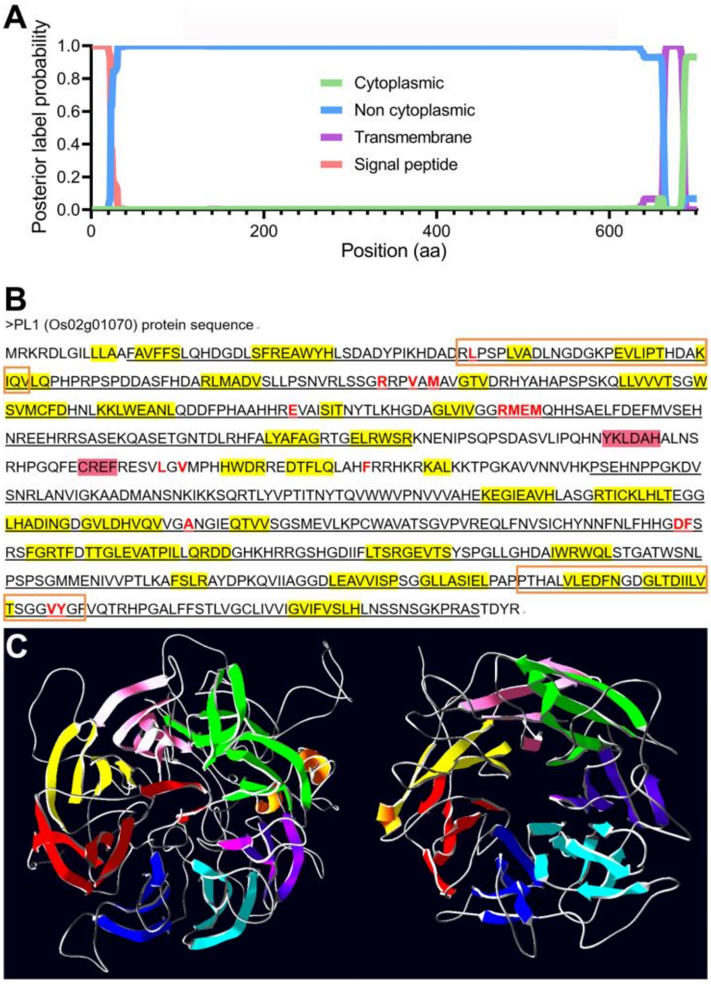

Figure S8. Bioinformatics analysis of PL1 protein. (**A**) the signal peptide, cytoplasmic region, transmembrane region and non-cytoplasmic region of PL1 predicted by TMHMM 2.0. (**B**) protein sequence of PL1 FG-GAP repeat domains determined by SMART are outlined by orange frames. β-stands are highlighted by yellow background. α-helixes are highlighted by pink background. Ca^{2+}-binding residues predicted by RaptorX are in red color. (**C**) Comparison of 3D structures models of PL1 (right) and human integrin α4-subunit (left). The N-terminal (from 35 aa to 478 aa) of Integrin α4-subunit (GenBank ID AAB59613.1) and full-length of PL1 were used for modeling by employing RaptorX

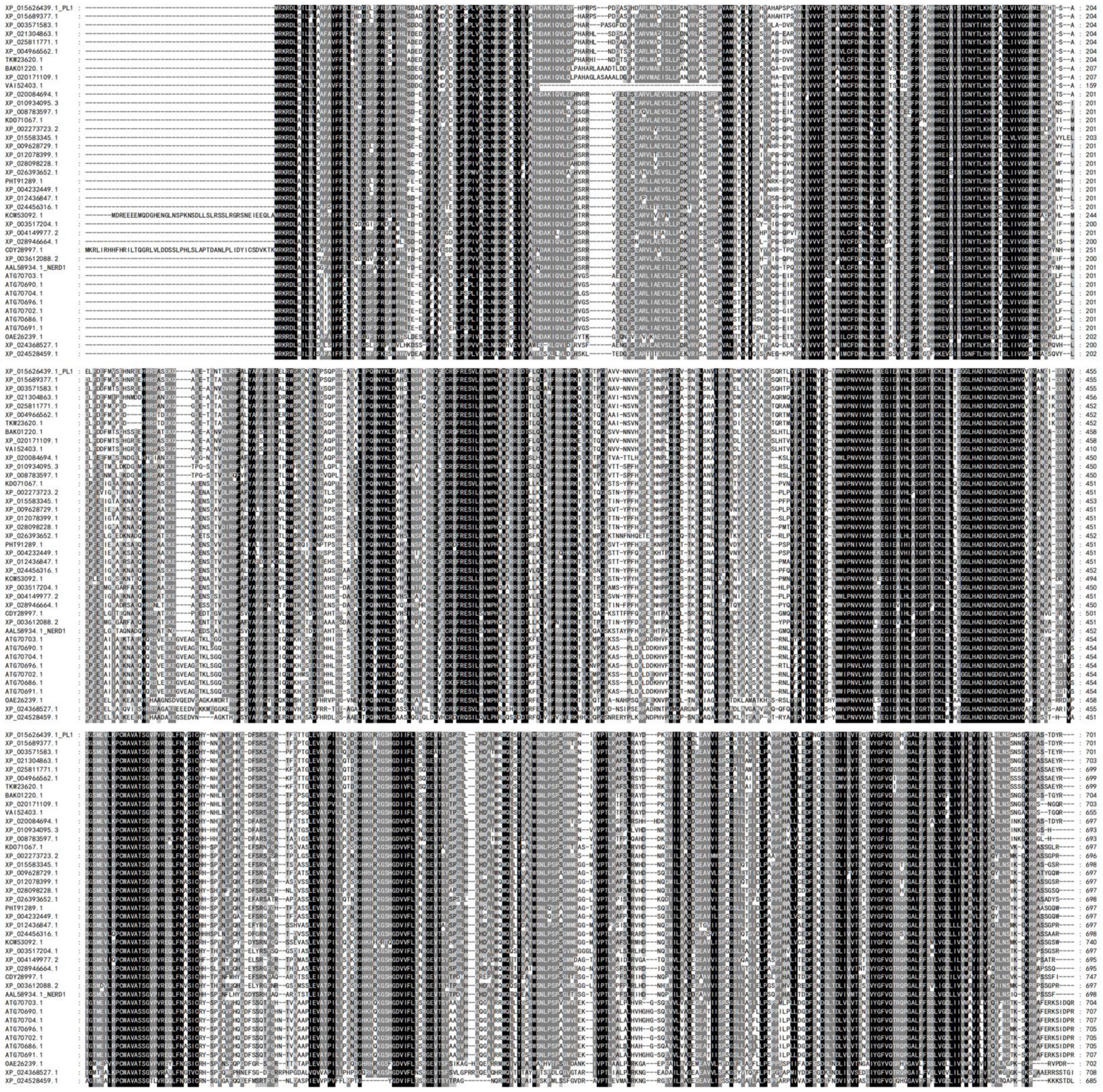

Figure S9. Protein sequences alignment of PL1 and the related homologs.

Table S1. ***pl1*** **segregation in F_2 populations.**

Phenotype or genotype	Expected	Observed	χ^2	*p* value
Normal-fertility; Male-sterility	3:1	207:60	0.4713	0.4924
PL1/PL1; PL1/pl1; pl1/pl1	1:2:1	64:143:60	0.7437	0.6895

Table S2. Phenotypic and genotypic association analysis of ***ko*** **plants progeny.**

Line	Fertility	Homo-Nipp [a]	Heterozygous	Biallelic or Homo-MT [b]
ko-1-1	Normal fertility	9	20	0
	Male sterility	0	0	31
ko-1-2	Normal fertility	6	12	0
	Male sterility	0	0	46
ko-2-1	Normal fertility	10	16	0
	Male sterility	0	0	38
ko-2-2	Normal fertility	18	8	0
	Male sterility	0	0	41

The numbers in this table indicate the numbers of plants with corresponding genotypes and phenotypes. [a] indicate homozygous *PL1* genotype; [b] indicate biallelic or homozygous mutation.

Table S3. Ratio of each mRNA species in plants with different genotype.

Transcript variants	*PL1/PL1*	*PL1/pl1*	*pl1/pl1*
PL1	100.00%	56.48%	0.00%
pl1.1	0.00%	25.00%	30.41%
pl1.2	0.00%	10.19%	44.59%
pl1.3	0.00%	6.48%	14.86%
pl1.4	0.00%	1.85%	10.14%

Table S4. Information of PL1-related proteins identified using BLAST.

GenBank IDs	Descriptions	Species	Identities (%)	e-values	Alignment length (aa)
XP_015626439.1	uncharacterized protein LOC4327975	*Oryza sativa*	100.00	0.0	701
XP_015689377.1	PREDICTED: uncharacterized protein LOC102718059	*Oryza brachyantha*	96.43	0.0	701
XP_003571583.1	uncharacterized protein LOC100827170	*Brachypodium distachyon*	88.18	0.0	702
XP_021304863.1	uncharacterized protein LOC110430941	*Sorghum bicolor*	85.51	0.0	704
XP_025811771.1	uncharacterized protein LOC112889389	*Panicum hallii*	85.09	0.0	704
XP_004966562.1	uncharacterized protein LOC101764188	*Setaria italica*	84.80	0.0	704
TKW23620.1	hypothetical protein SEVIR_4G303200v2	*Setaria viridis*	84.80	0.0	704
BAK01220.1	predicted protein	*Hordeum vulgare*	84.26	0.0	705
XP_020171109.1	uncharacterized protein LOC109756672	*Aegilops tauschii*	83.69	0.0	701
VAI52403.1	unnamed protein product	*Triticum turgidum*	80.03	0.0	697
XP_020084694.1	uncharacterized protein LOC109707659	*Ananas comosus*	79.94	0.0	703
XP_010934095.3	uncharacterized protein LOC105054295	*Elaeis guineensis*	76.35	0.0	698
XP_008783597.1	uncharacterized protein LOC103702808	*Phoenix dactylifera*	75.78	0.0	697
KDO71067.1	hypothetical protein CISIN_1g005433mg	*Citrus sinensis*	74.57	0.0	704
XP_002273723.2	PREDICTED: uncharacterized protein LOC100264247	*Vitis vinifera*	74.40	0.0	703
XP_015583345.1	uncharacterized protein LOC8273019	*Ricinus communis*	73.65	0.0	702
XP_009628729.1	PREDICTED: uncharacterized protein LOC104119039	*Nicotiana tomentosiformis*	73.01	0.0	700
XP_012078399.1	uncharacterized protein LOC105639070	*Jatropha curcas*	73.44	0.0	704
XP_028098228.1	uncharacterized protein LOC114297952	*Camellia sinensis*	73.72	0.0	701
XP_026393652.1	uncharacterized protein LOC113288746	*Papaver somniferum*	72.62	0.0	704
PHT91289.1	hypothetical protein T459_06402	*Capsicum annuum*	72.59	0.0	701
XP_004232449.1	uncharacterized protein LOC101246491	*Solanum lycopersicum*	72.16	0.0	701
XP_012436847.1	PREDICTED: uncharacterized protein LOC105763250	*Gossypium raimondii*	71.73	0.0	704
XP_024456316.1	uncharacterized protein LOC7468882	*Populus trichocarpa*	71.81	0.0	706
KCW53092.1	hypothetical protein EUGRSUZ_J02388	*Eucalyptus grandis*	65.73	0.0	701
XP_003517204.1	uncharacterized protein LOC100787497	*Glycine max*	71.06	0.0	705
XP_004149977.2	PREDICTED: uncharacterized protein LOC101223217	*Cucumis sativus*	71.73	0.0	700
XP_028946664.1	uncharacterized protein LOC103401057	*Malus domestica*	69.46	0.0	699
CDY28997.1	BnaC07g32030D	*Brassica napus*	63.53	0.0	703
XP_003612088.2	uncharacterized protein LOC11412168	*Medicago truncatula*	69.79	0.0	705
AAL58934.1	AT3g51050/F24M12_90	*Arabidopsis thaliana*	67.42	0.0	705
ATG70703.1	FG-GAP repeat-containing protein	*Thuja plicata*	63.43	0.0	703
ATG70690.1	FG-GAP repeat-containing protein	*Cupressus sempervirens*	63.45	0.0	706
ATG70704.1	FG-GAP repeat-containing protein	*Callitropsis nootkatensis*	63.17	0.0	706
ATG70696.1	FG-GAP repeat-containing protein	*Juniperus indica*	62.78	0.0	704
ATG70702.1	FG-GAP repeat-containing protein	*Microbiota decussata*	63.2	0.0	704
ATG70686.1	FG-GAP repeat-containing protein	*Calocedrus decurrens*	63.48	0.0	704
ATG70691.1	FG-GAP repeat-containing protein	*Hesperocyparis arizonica*	62.89	0.0	706
OAE26239.1	hypothetical protein AXG93_3786s1190	*Marchantia polymorpha*	57.40	0.0	703
XP_024368527.1	uncharacterized protein LOC112278889	*Physcomitrella patens*	54.05	0.0	708
XP_024528459.1	uncharacterized protein LOC9632834	*Selaginella moellendorffii*	52.91	0.0	703

Table S5. Primers sequences used in this study.

Primer	Forward primer	Reverse primer	Annotation
SEQ-P	GGAAGAGCACCGTAGAAG	GAGTCAAGACAGCCACCT	Sequencing of the *pl1* mutation
SEQ-K	GCCTTCGCCGTCTTCTTC	TTGCGTAGCCTTGCCTGA	Sequencing of the CRISPR/Cas9 targets
SEQ-1	CAGGTTTGGTCATCGTCGGAG	N/A	Sequencing
SEQ-2	GAAAAAGAAGGGATAGAGGCTGT	N/A	Sequencing
KO-1	ggcaAGAGGTGGTACCAGGCCTCG	aaacCGAGGCCTGGTACCACCTCT	CRISPR/Cas9 target 1
KO-2	ggcaGAGGAGAGGCGAACGTTGGA	aaacTCCAACGTTCGCCTCTCCTC	CRISPR/Cas9 target 2
Set-FL	ATGCGGAAGCGGGATCTG	TCACCTATAGTCGGTTGAAGCC	RT-PCR and sequencing
Set-A	CGTCATCCTGGTCAGTTCG	GCAGCTTTCCCAATCACAT	RT-PCR
Set-B	CTCATGGCTGATGTCTCCCT	CGGTGCTCTTCCCTGTTG	RT-PCR
Set-C	GAAGAGCACCGTAGAAGCG	CCTATCCCAATGATGAGGC	RT-PCR and sequencing
qPL1	CTTCGACCACAACCTCAAAAAG	AGTTGGTAATGGAAATGGCAAC	qRT-PCR
qUbi	CAAGATGATCTGCCGCAAATGC	TTTAACCAGTCCATGAACCCG	qRT-PCR
qCYP703A3	GGCAAGATGAGGTATTTGCTTC	GGTGAGCAGCAAAAGATTCAAG	qRT-PCR
qOsACOS12	GAGGAGGAGATAGTGGCGTA	TCTTGATGAACTCGTCCCTAAG	qRT-PCR
qOsPKS2	GAGAAGCTCACCAGACTCTG	GTAGACAAGGTGGGTGATCTC	qRT-PCR
qOsTKPR1	TTCTAAACCGAATCTTGTTGCG	TTATGAGGGCAGTTCGTTGATC	qRT-PCR
qOsSTRL2	CTTGAAGTAGAGCGTTCGCAG	GACAACGTGCGACTGAGCA	qRT-PCR
qOsABCG26	GATACAGCTCAATTCTGGCAAG	ATCGTGTTGCTTATCACGAATG	qRT-PCR
qOsNP1	TGAAGATTCAGTCCGAGAGATG	TCAACAAGCAGATACTGAACCT	qRT-PCR
qEAT1	CTGAAAAGAAGAAGGCCAACTC	CACCTATAATCCCACCCACTAC	qRT-PCR
qPTC1	GTTCCCGTCCTTCAACTGTTAG	AGCATCATATATAGCAGCAGAGG	qRT-PCR
GUS-PL1	aaaaaagcaggctccgaattcCGACCCAG CGTGAGGGAA	caagaaagctgggtcgaattcCTCGCCGCTCT CTCTCTCTCT	Promoter-GUS assay
YFP-PL1	accagtctctctcaagcttATGCGGAAG CGGGATCTG	catactagtggatccaagcttCCTATAGTCGGT TGAAGCCCTAGG	Subcellular location

The construction adapter is in lower character.

Table S6. Information of templates used to arrive at PL1 model by RaptorX.

Template(s)	*P*-value [a]	Score [b]	uGDT/GDT [c]
1kv9A	5.50e-07	128	157/22
1kb0A	3.50e-07	131	150/21
4mh1A	1.40e-06	120	142/20
1yiqA	2.60e-07	134	141/20
4hdjA	2.50e-07	134	142/20

[a], *P*-value is the likelihood of a predicted model being worse than the best of a set of randomly generated models for this protein (or domain). [b], score is the alignment score falling between 0 and the (domain) sequence length, with 0 indicating the worst. [c], uGDT is the unnormalized Global Distance Test score defined as 1*N(1)+0.75*N(2)+0.5*N(4)+0.25*N(8), where N(x) is the number of residues with estimated modeling error (in Å) smaller than x. GDT is calculated as uGDT divided by the protein (or domain) length and multiplied by a 100. uGDT(GDT) measures the absolute model quality.
